# A novel c.2179T>C mutation blocked the intracellular transport of *PHEX* protein and caused X‐linked hypophosphatemic rickets in a Chinese family

**DOI:** 10.1002/mgg3.1262

**Published:** 2020-06-08

**Authors:** Baowei Li, Xiong Wang, Xiaodan Hao, Yanran Liu, Yin Wang, Chan Shan, Xiang Ao, Ying Liu, HongChu Bao, Peifeng Li

**Affiliations:** ^1^ Institute for Translational Medicine Qingdao University Qingdao China; ^2^ Department of Reproductive Medicine Affiliated Yantai Yuhuangding Hospital of Qingdao University Yantai China; ^3^ School of Basic Medicine Qingdao University Qingdao China

**Keywords:** glycosylation, *PHEX*, whole‐exome sequencing (WES), X‐Linked hypophosphatemic rickets (XLH)

## Abstract

**Background:**

X‐linked hypophosphatemic rickets (XLH) is a heterogeneous genetic phosphate wasting disorder that occupies the majority of inheritable hypophosphatemic rickets (HR). XLH is caused by loss‐of‐function mutations in the phosphate‐regulating endopeptidase gene (*PHEX*) located on the X chromosome.

**Method:**

In this study, we performed whole‐exome sequencing (WES) on the proband to identify the causative gene. The mutations were analyzed by predictive online software, such as PolyPhen‐2. Plasmids containing the wild‐type (WT) and mutant cDNA of the candidate gene were transfected into HEK293, then, the expression, cellular localization, and glycosylation state of the candidate proteins were detected by western blot, immunostaining, and endoglycosidase H digestion. The expression and concentration of related factor were measured by RT‐PCR and ELISA.

**Results:**

We identified a novel missense mutation c.2179T>C in the *PHEX* that results in the substitution of p.Phe727Leu (F727L). This mutation was predicted to be disease‐causing by all four predictive online software. In vitro studies demonstrated that the F727L substitution hindered the intracellular trafficking of the mutant PHEX, with ~59% of mutant PHEX protein retained in the endoplasmic reticulum (ER) and only ~16% of the mutant protein localized on the cell surface. Endoglycosidase H digestion assay showed that the mutant F727L PHEX protein was not fully glycosylated. The concentration of intact FGF23 in hFOB1.19 cell culture medium collected from the mutant PHEX group was the highest (62.9 pg/ml) compared to the WT group (32.1 pg/ml) and control group (23.5 pg/ml).

**Conclusion:**

Our results confirmed that the mutant PHEX protein was lowly glycosylated and retarded within the ER, the intact FGF23 level in cell culture media caused by the mutant PHEX protein was significantly elevated compared to that of the WT group, which may explain why the single base mutation in the *PHEX* led to XLH syndrome in this family.

## Graphical abstract

In this study, we identified a novel missense mutation c.2179T>C (p.F727L) in the *PHEX* from an XLH family. We demonstrated that the F727L mutation decreased the glycosylation level of PHEX protein, blocked PHEX protein transport from the ER to the plasma membrane, and reduced its activity to cleave FGF23 into inactive segments, which may explain why the single base substitution c.2179T>C led to the hypophosphatemic rickets in this Chinese family.

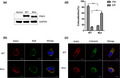

## 1 INTRODUCTION

Hypophosphatemic rickets (HR) are a group of abnormal skeletal mineralization diseases caused by defective phosphate reabsorption in the proximal renal tubules (Rowe, 1994, 1998; Yue et al., 2014). There are four main subtypes of HR: X‐linked dominant hypophosphatemia (XLH), autosomal recessive hypophosphatemic rickets (ARHR) and autosomal dominant hypophosphatemic rickets (ADHR), and tumor‐induced osteomalacia (TIO) (Dhir, Li, Hakonarson, & Levine, 2017; Drezner, 2000; Kapelari, Kohle, Kotzot, & Hogler, 2015; Pal et al., 2019; Quarles & Drezner, 2001). XLH (MIM 307800) is the main form of heritable HR, causing 87% of familial HR cases (Quinlan et al., 2012), with an occurrence of approximately 1 in 20,000 live births (Chandran et al., 2010). XLH is caused by loss‐of‐function mutations in the phosphate‐regulating endopeptidase gene (*PHEX*, OMIM No. 300550; Fuente et al., 2017) and is characterized by vitamin D resistance, hypophosphatemia, growth retardation, bone malformations, short stature, and dental abnormalities (Durmaz et al., 2013; Fuente et al., 2017; Ruppe et al., 2011).

The *PHEX* consists of 22 exons that reside on chromosome Xp22.1 to 22.2 that encode a transmembrane glycoprotein composed of 749 amino acids (Clausmeyer et al., 2009; Lipman et al., 1998). The PHEX protein consists of a short N‐terminal cytoplasmic region, a single N‐terminal hydrophobic transmembrane region, and a large extracellular C‐terminal domain (Sabbagh, Boileau, DesGroseillers, & Tenenhouse, 2001). This protein belongs to the type II integral membrane zinc‐dependent endopeptidase family and functions as an extramembrane endopeptidase (Lipman et al., 1998). It can directly or indirectly cleave FGF23, a phosphate‐regulating hormone expressed in osteocytes (Quarles, 2012), into an N‐terminal segment and a C‐terminal segment, both of which lose the ability to inhibit renal tubular phosphate transport and facilitate the maintenance of blood phosphate (Bowe et al., 2001; Yamazaki et al., 2002). Inactivating mutations in PHEX lead to the accumulation of intact circulating FGF23, which decreases phosphate reabsorption in renal tubules and causes abnormal bone mineralization (Sabbagh, Boileau, Campos, Carmona, & Tenenhouse, 2003).

Although PHEX plays a vital role in phosphate reabsorption in the renal tissue, it is mainly expressed in osteocytes, osteoblasts, and odontoblasts but not in any renal tissues (Guo & Quarles, 1997; Sabbagh et al., 2003). Currently, 523 different PHEX variations have been reported in the ClinVar database, of which 369 variations are pathogenic. However, the mechanisms of the pathogenesis of XLH of most of these variations have not been elucidated. In this study, a novel missense mutation (c.2179T>C) in the *PHEX* was identified in a Chinese family with XLH (den Dunnen et al., 2016). Molecular cytobiological analysis showed that this mutation altered PHEX protein structure, glycosylation level, and cellular localization. These findings increased the *PHEX* mutation spectrum and provided a molecular biological basis for the diagnosis of HR.

## 2 MATERIALS AND METHODS

### 2.1 Subjects and ethical statement

This study was approved by the Ethics Committee of the Affiliated Yantai Yuhuangding Hospital of Qingdao University (Yantai, China), and all subjects (five members of this XLH family and 200 normal individuals) provided written informed consent prior to enrollment. All of the blood samples, clinical data, and X‐ray photos were collected at the Affiliated Yantai Yuhuangding Hospital of Qingdao University. This project was conducted following the Declaration of Helsinki.

### 2.2 Genomic DNA isolation

Genomic DNA was isolated from peripheral whole blood samples (trisodium citrate) using the Gentra Puregene Blood Kit (Qiagen Corp) according to the manufacturer's instructions. The isolated DNAs were stored at −20°C until use.

### 2.3 Whole‐exome sequencing

The DNA of the proband (III‐2) was subjected to whole‐exome sequencing (WES) analysis (Hyde Source Biotech). Exomes were enriched with the Agilent SureSelect Human All Exon V5 Kit and sequenced on an Illumina NextSeq500 platform. The raw data were mapped to the reference genome (hg19) by Burrows Wheeler Aligner (BWA) software. Genome variation was identified by Picard software and then annotated by ANNOVAR software. The reference database includes the normal human genome databases: 1,000 Genomes (https://www.ncbi.nlm.nih.gov/variation/tools/1000genomes/), dbSNP (https://www.ncbi.nlm.nih.gov/snp/); and the disease databases: OMIM (https://www.ncbi.nlm.nih.gov/omim), the Human Gene Mutation Database (http://www.hgmd.cf.ac.uk/ac/index.php), and ClinVar (http://www.ncbi.nlm.nih.gov/clinvar/). To screen for possible pathogenic mutations, we filtered out MAF (>1%) mutations in the 1,000 Genomes and dbSNP databases and screened pathogenic information with the ACMG Standards and Guidelines. For the candidate pathogenic mutation, we used PolyPhen‐2, Mutation Taster, PROVEAN, and SIFT software to predict the mutational consequence of PHEX (protein identifier #P78562) (Adzhubei et al., 2010; Feng, 2017).

### 2.4 Sanger sequencing

The nucleotide sequence of the *PHEX* (NC_000023.11, NM_000444.6) was acquired from the National Center for Biotechnology Information (NCBI). The specific primers flanking the mutant site were designed using Primer Premier version 5 software. Forward primer (F) 5′‐AAGAGTAATAGGGGCATGTGCTTG‐3′; reverse primer (R) 5′‐TCTGTTCATCGTGGAATTGGGT‐3′; sequencing primer 5′‐CAGGAGATGCTGGATCTAGTCTGTTA‐3′. Polymerase chain reaction (PCR) was performed to amplify a 297‐bp product containing the mutant site. The 297‐bp products were used as templates in the dideoxy chain‐termination PCR system using BigDye Terminator v3.1 Kit (Applied Biosystems), and the sequence data were obtained from the 3730xl DNA analyzer (Applied Biosystems).

### 2.5 Mutation analysis

Following Sanger sequencing of candidate pathogenic mutations in all members of this family, we analyzed the conservativeness of the mutant site among different species using ClustalW and predicted the changes in protein structure with SWISS‐MODEL and PyMOL software (Hu, Hua, Chen, Yu, & Gao, 2015).

### 2.6 Plasmid construction

Human wild‐type PHEX cDNA was cloned from hFOB1.19 cells and subcloned into plasmid pcDNA3.1(+) (Invitrogen), which contained the human cytomegalovirus (CMV) promoter and a flag‐tag sequence. PHEX missense mutations, c.2179T>C p.(F727L), identified in XLH patients were generated by PCR‐mutagenesis. The primers for cloning of whole‐length (2,250 bp) wild‐type PHEX cDNA were F 5′‐ATGGAAGCAGAAACAGGGAGC‐3′ and R 5′‐CTACCAGAGTCGGCAGGAGTCC‐3′. Primers for mutation of c.2179T>C p.(F727L) were F 5′‐AGTAACTTTGAAGAA***C***TCCAGAAAGCTTTTAACTGTCCACC‐3′, and R 5′‐TAAAAGCTTTCTGGA***G***TTCTTCAAAGTTACTAATTGCACCA‐3′. The identities of WT PHEX and F727L‐PHEX plasmids were confirmed by Sanger sequencing (Tsingke).

### 2.7 ELISA

FGF23 levels in serum and cell culture samples were measured using a human FGF23 enzyme‐linked immunosorbent assay (ELISA) kit that detects only intact FGF23 (Kainos Laboratories, Inc.). Blood samples from all two XLH patients (III‐2, III‐1), three normal individuals of this pedigree (II‐1, II‐2, and III‐1), and 100 normal individuals were collected and analyzed for intact FGF23. The cell culture supernatants were also analyzed for intact FGF23, which were collected from hFOB1.19 cells 48 hr after transfection with WT PHEX, Mut‐PHEX, and blank vectors. Briefly, samples were incubated with the immobilized anti‐FGF23 N‐terminal antibody in a microtiter well. Wells were washed to remove unbound FGF‐23 and other components. Then, the immobilized FGF23 is incubated with HRP‐labeled FGF23 C‐terminal antibody to form a “sandwich complex.” The sandwich complex is incubated with a substrate solution, and then the enzymatic activity of the complex is measured by a spectrophotometric microtiter plate reader. The absorbance of OD450 directly represents the amount of FGF23 in the sample.

### 2.8 Cell culture and transfection

HEK293 cells were cultured in DMEM (Sangon Biotech) with 10% fetal bovine serum (FBS) (Sangon Biotech) and maintained in a 37°C humidified incubator with 5% CO_2_. HFOB1.19 cells, obtained from Cell Bank of Chinese Academy of Sciences, were cultured in DMEM/F12 (Sangon Biotech) containing 10% fetal bovine serum (FBS) (Sangon Biotech) and 0.3 mg/ml G418 and maintained in a 33.5°C humidified incubator with 5% CO2. HEK293 cells were split into 6‐well plates containing four 8 mm × 8 mm coverslips per well. Cells were transiently transfected using Lipofectamine 3000 reagent (Invitrogen) according to the manufacturer's protocol and plasmids encoding either the WT PHEX or F727L‐PHEX or an empty pcDNA3.1(+) vector. HEK293 cells were lysed for western blotting or fixed for immunofluorescence staining 48 hr after transfections. HFOB1.19 cells were split into 60 mm dishes and transfected with plasmids encoding either the WT PHEX or F727L‐PHEX or an empty pcDNA3.1(+) vector using Lipofectamine 3000. HFOB1.19 cells were lysed for western blotting 48 hr after transfections or real‐time quantitative PCR (qPCR) analysis 24 hr after transfections. When the cells were transfected for 24 hr, the media of hFOB1.19 cells were changed with FBS‐free DMEM/F12 and maintained in the incubator for another 24 hr. Then, the media were collected for detection of FGF23 levels by ELISA. The media of HEK293 cells were also changed with FBS‐free DMEM 24 hr after transfection and maintained in the incubator for another 24 hr to facilitate PHEX protein secretion.

### 2.9 Western blotting

Transfected HEK293 cells and hFOB1.19 cells were lysed using radio immunoprecipitation assay (RIPA) buffer containing the protease inhibitor and PMSF. Secreted proteins in FBS‐free DMEM supernatants of HEK293 cells were concentrated using the TCA Protein Precipitation Kit (Sangon Biotech) and redissolved in 1 × denature protein loading buffer. Proteins were separated through 12% SDS–polyacrylamide gels and transferred to 0.22 μm nitrocellulose filter membranes. A rabbit anti‐PHEX polyclonal antibody (Sigma‐Aldrich, SAB2700899) was used at a 1:2000 dilution, and a rabbit anti‐ FGF23 polyclonal antibody (Abclonal, A6124) was used at a 1:1,000 dilution. Secondary HRP‐conjugated goat anti‐rabbit antibody (Sangon Biotech) was used at a 1:10,000 dilution. The signals of antibody immune complexes were captured by the FUSION Solo S imaging system (Vilber Lourmat) using the BeyoECL Star chemiluminescence Kit (Beyotime Biotech).

### 2.10 Reverse transcription polymerase chain reaction

Total RNA from hFOB1.19 cells was extracted using TRIzol reagent (Sangon Biotech) according to the manufacturer's protocol. A total of 500 ng of RNA was reverse‐transcribed into cDNA using SuperScript III transcriptase (Invitrogen). PCR was performed with 1 × PrimeSTAR Mix (TaKaRa) to generate WT PHEX DNA. The cycle conditions were as follows: 95 for 3 min followed by 35 cycles (98°C for 10 s, 62°C for 30 s, 72°C for 20 s), with a final incubation at 72°C for 2 min. The qPCR cycle conditions were as follows: 95 for 3 min followed by 40 cycles (98℃ for 10 s, 58℃ for 30 s, 72℃ for 20 s), signals were acquired after extension at 72℃ in each cycle. Primers for qPCR were as follows, GAPDH: F 5′‐CTGACTTCAACAGCGACACC‐3′ and R 5′‐TGCTGTAGCCAAATTCGTTGT‐3′; FGF23: F 5′‐CGACGTCTACCACTCTCCTC‐3′′ and R 5′‐TGGTATGGGGGTGTTGAAGT‐3′.

### 2.11 Cellular localization analysis of PHEX protein expression

Transfected HEK293 cells were fixed in PBS containing 4% paraformaldehyde, and immunofluorescence staining was performed as described (Li et al., 2011). To assess the localization of PHEX at the endoplasmic reticulum (ER), cells were permeabilized with 0.1% Triton X‐100 for 10 min. Nonpermeabilized cells were used to assess the localization of PHEX at the cellular membrane. Immunostaining was performed using primary antibodies, including anti‐Flag (a tag fused with PHEX)(1:500; AF519(M) or AF0036(R); Beyotime Biotech), anti‐Na‐K‐ATPase (1:200; AF1864(R), Beyotime Biotech), and anti‐calnexin (1:200; AC018(M), Beyotime Biotech) antibodies, and secondary antibodies, including goat anti‐mouse AlexaFluor‐488, goat anti‐mouse AlexaFluor‐555, and goat anti‐rabbit AlexaFluor‐555 antibodies (Sangon Biotech). Images were captured using a Leica TCS SP8 confocal microscope. The colocalization proportion was calculated using ImageJ software. The percentage of PHEX signals at the plasma membrane (PM) or ER was quantified using a minimum of five slides from at least three separate experiments.

### 2.12 Endoglycosidase H digestion

Cell lysate samples and TCA extracted cell media containing recombinant PHEX proteins generated by transient transfected 293 cells were treated with endoglycosidase H (Endo H) to detect the glycosylation state of the proteins. Briefly, 20 µg of total cell proteins of cell lysate and 30 μl of concentrates from 30 ml cell media in 1× glycoprotein‐denature buffer was boiled at 100℃ for 10 min and then incubated in 1× reaction buffer with Endo H at 37℃ for 60 min. Endo H‐treated and untreated samples were analyzed by electrophoresis and western blotting.

### 2.13 Data analyses

Unpaired Student's *t* tests were used in data comparison between groups. Differences were considered to be statistically significant when the values of *p* < .05. Data analyses were performed using SPSS 16.0 software.

## 3 RESULTS

### 3.1 Case report

**Figure 1:**
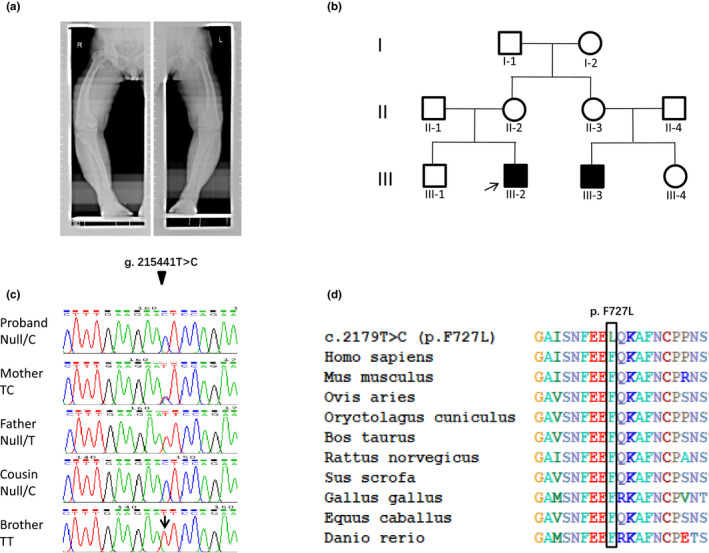
The radiology results of the patients, pedigree, and mutation analysis of this family with XLH. (a) The proband had obviously bent long bones in the lower limbs and presented severe genu varum deformities. (b) Filled‐in shapes represent the affected individuals, and unfilled drawings represent the unaffected individuals. Circles and squares represent the females and males, respectively. The proband is indicated by the black arrow. (c) Partial DNA sequencing in exon 22 on the *PHEX* (NC_000023.11) showed the individual genotypes at the g.215441T/C locus, which is indicated by black arrows. At this locus, the proband was a mutant hemizygote (Null/C), his mother was a heterozygote (TC), his father and brother were both WT homozygotes (TT), and the son of his mother’s sister was a mutant hemizygote (Null/C). (D) Multiple alignment of PHEX from ten different species showed that the F727L substitution occurred in a highly conserved position, which is framed by a black rectangle

On October 9, 2017, a 38‐year‐old man came to the Department of Reproductive Medicine for fertility problems. The man had obvious developmental defects. He was only 152 cm high, with a forward waist, and severe genu varus (Figure 1a), but he did not suffer any obvious arthrodynia. When asked about the rest of his family, we learned that a son of his mother's sister has a similar symptom. The affected individuals were indicated in the pedigree of this family (Figure 1b). To ensure the real reason for the proband's syndrome, we made a preliminary diagnosis of his disease, including the bone mineral density (BMD) test and serum biochemical test. The results from dual energy X‐ray absorptiometry (DEXA) showed that the BMD of lumbar vertebrae (L1–L3) was 1.576 g/cm^2^, and the *T* value and the *Z* value are both in the normal range compared with the peak bone density of the same sex and with the BMD of contemporaries, respectively (Fig. S1a,b). The BMD values of the left and right femoral neck were 1.252 and 1.112 g/cm^2^, respectively, and the *T* value and the *Z* value are both in the normal range (Fig. S1c,d). More detailed BMD data are shown in Table S1, and all these data indicate that the proband's BMD is in the normal range. Biochemical tests showed that the proband has a reduced blood phosphate concentration (1.76 mg/dl), elevated parathyroid hormone (PTH; 84.44 pg/ml), and normal 25(OH)D (16.3 ng/ml; Table 1). The son of his mother's sister suffered similar symptoms, with a height of 155 cm and a reduced blood phosphate concentration (1.65 mg/dl), elevated PTH (87.36 pg/ml), and normal 25(OH)D (18.1 ng/ml; Table 1). His mother presents a mild genu varus with a height of 157 cm, and his father and elder brother are both normal and have no HR symptoms (Table 1). Based on these data, the disease of the proband was initially diagnosed as hypophosphatemia.

**Table 1:** Clinical data and biochemical results

ID	Gender	Age (y)	Height (cm)	Weight (kg)	Clinical symptoms	Serum P (mg/dL)	ALP (u/L)	PTH (pg/mL)	25(OH)D(ng/mL)
II‐1	Male	68	174	74	Normal	2.96.	126	46.24	21.6.
II‐2	Female	66	157	65	Mild genu varus	1.92↓	131	94.89↑	24.1
III‐1	Male	41	175	85	Normal	3.12	115	32.56.	20.7
III−2	Male	38	152	75	Severe genu varus	1.76↓	84	84.44↑	16.3
III−3	Male	42	155	89	Severe genu varus	1.65↓	92	87.36↑	18.1

Reference: serum P, 2.5‐4.5 mg/dl; ALP, 50‐135 U/L (female), 45‐125 U/L (male); PTH, 12‐65 pg/ml; 25(OH)VD, 8‐50 ng/ml.

### 3.2 Identification of the mutation locus in the proband

The exon regions of patients were detected by WES. The average depth of sequencing was 67×, and the average coverage was 96.8%. Compared with dbSNP, the 1,000 Genomes Project and other databases, we found that there was a novel missense mutation g.215441T>C (c.2179T>C; p.F727L) in exon 22 of the *PHEX* on chromosome X (NC_000023.11), and this gene is related to hypophosphatemia.

### 3.3 Mutation analysis and protein modeling

Sanger sequencing results showed that the proband was a mutant hemizygote in this locus, the proband's mother was a heterozygote, his father and brother had no mutation in this locus, and the son of his mother's sister was also a mutant hemizygote, so the mutation of this locus in the proband was inherited from his mother (Figure 1c). Two hundred healthy individuals had no mutations in this locus detected by Sanger sequencing (data not shown). The above results indicate that the g.215441T>C mutation is coseparated from the disease phenotype in this family, and it is suggested that this mutation is a pathogenic mutation in the XLH family. Mutation Taster, Poly Phen‐2, PROVEAN and SIFT online software were used to predict and analyze the effect of the F727L mutation on the function of the *PHEX*. The results showed that the mutation was predicted to be damaging by all four software packages: SIFT (score: 0), Mutation Taster (*p* value: 1), PROVEAN (score: −5.562) and Poly Phen‐2 (score: 1).

We then analyzed the amino acid conservation of PHEX p.F727 residue between different species by ClustalW. The results showed that the phenylalanine residue at p.F727 is highly conserved across 10 vertebrate species (Figure 1d). The physicochemical properties of amino acids at the p.F727L mutation site were analyzed. We found that the F727L mutation resulted in the replacement of the aromatic amino acid phenylalanine with the aliphatic amino acid leucine.

**Figure 2:**
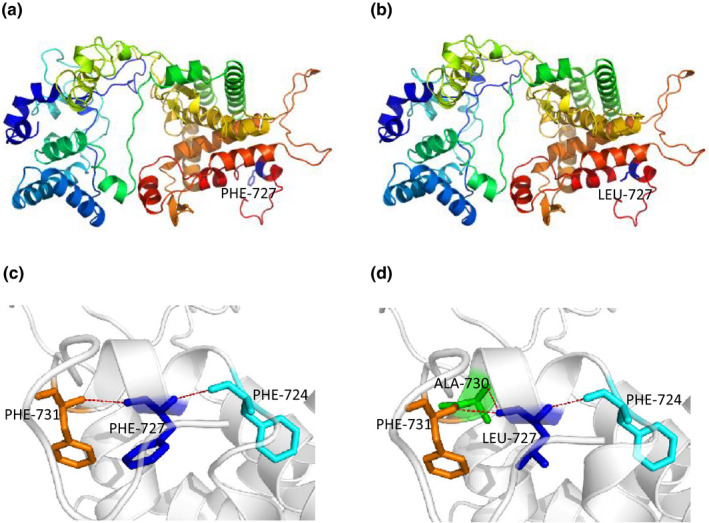
Model structure of the p.F727L mutation. (a and b) The three‐dimensional model of WT and mutant F727L PHEX protein was generated by SWISS‐MODEL and analyzed by PyMOL software. In the WT protein, the PHE residue at position 727 presented a loose local structure (a), and in the mutant protein, the LEU residue at position 727 presented a tight local structure (b). (c and d) Detailed structure analysis of hydrogen bonds of F727 and 727L. The mutant Leu residue added one more hydrogen bond to residue ALA730 (c) compared to the WT PHE residue, which only has two hydrogen bonds to residues PHE724 and PHE731 (d). These structures are shown on the same scale and orientation. The PHE and LEU residues at position 727 are blue in the model structures. The correlated residues are presented by sticks, and the hydrogen bonds are indicated by brown dotted lines

Protein modeling showed that the mutated residue leucine reduced the space around itself (Figure 2a) and added a new hydrogen bond with Ala730 (Figure 2c) compared to the WT‐ F727 residue, which has a locally looser space (Figure 2b) and two hydrogen bonds with Phe724 and Phe731 (Figure 2d). These results indicated that the F727L mutation likely caused changes in protein structure and function.

### 3.4 Serum FGF23 levels were elevated in XLH patients

The reference range of intact serum FGF23 levels from 100 healthy individuals aged between 19 and 72 years was from 26.2 to 66.4 pg/ml (Table S2), and the median value was 46.2 pg/ml. The intact serum FGF23 levels of the proband and his elderly male cousin were 127.3 and 139.6, respectively (Table 2), which were much higher than the median value and exceeded the maximum reference value. The proband's mother has a marginally elevated FGF23 level with a value of 81.3 pg/ml, and his father has a normal FGF23 level with a value of 42.9 pg/ml (Table 2).

**Table 2:** Serum intact human FGF23 levels in the XLH family

Subjects	FGF23(pg/mL)	Subjects	FGF23 (pg/ml)
III‐2	127.3	II‐1	42.9
III‐3	139.6	II‐2	81.3

### 3.5 The F727L mutation altered protein location in transfected cells

**Figure 3:**
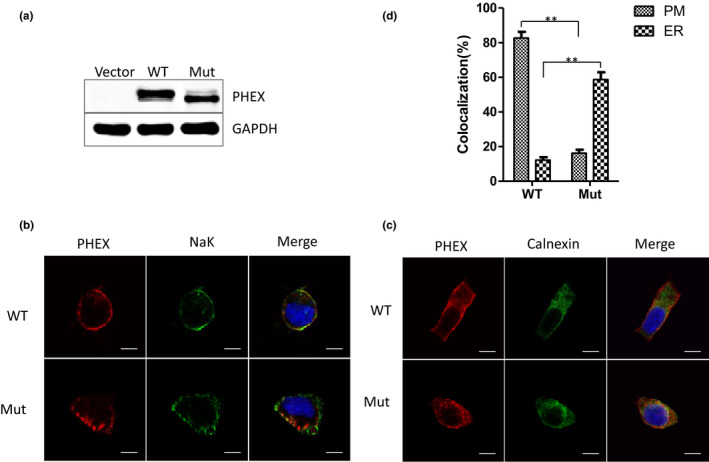
Expression and localization of WT and mutant F727L PHEX in HEK293. (a) Western blot analysis showed that WT and mutant F727L PHEX proteins were successfully expressed in HEK293 cells following transfection with a vector encoding WT or mutant F727L PHEX, and there was no PHEX expression in HEK293 cells transfected with an empty control vector. (b) Immunofluorescence staining was performed in nonpermeabilized HEK293 cells, and the localization of PHEX protein (red) at the plasma membrane (PM) was indicated by colocalization with Na‐K‐ATPase (green). (c) Immunofluorescence staining was performed in permeabilized HEK293 cells, and the localization of PHEX protein (red) at the ER was indicated by colocalization with calnexin (green). (d) Percentage of WT or mutant F727L PHEX protein that colocalized with PM or ER in HEK293 cells. Scale bars represent 10 μm. *p < .05; **p < .01. Bars show *SD* (*n* = 5)

To determine whether the F727L mutation affects the expression and cellular transport of PHEX, we transiently transfected WT (F727) or mutant (727L) PHEX coding vectors into HEK293 cells in vitro. Whole‐cell lysates were obtained from HEK293 cells transfected for 48 hr and were analyzed by western blot. The results showed that WT and mutant PHEX proteins were both elevated in the cells, while there was no PHEX expression in cells transfected with an empty pcDNA3.1(+) vector (control group) (Figure 3a). Immunofluorescence analysis of Triton X‐100‐treated (permeabilized) and Triton X‐100‐untreated (nonpermeabilized) cells was performed to detect the cellular localization of WT and mutant PHEX proteins (Figure 3b,c). Colocalization analysis of nonpermeabilized cells showed that ~83% of WT PHEX proteins were located at the PM, while only ~16% of mutant PHEX proteins were located at the PM (Figure 3d). In contrast, colocalization analysis of permeabilized cells revealed that only ~12% of WT PHEX was localized in the ER, and ~59% of mutant PHEX was associated with the ER (Figure 3d). These findings indicated that the F727L mutation impaired protein trafficking and retained the mutant PHEX protein in the ER.

### 3.6 Glycosylation level of WT and mutant PHEX proteins

**Figure 4:**
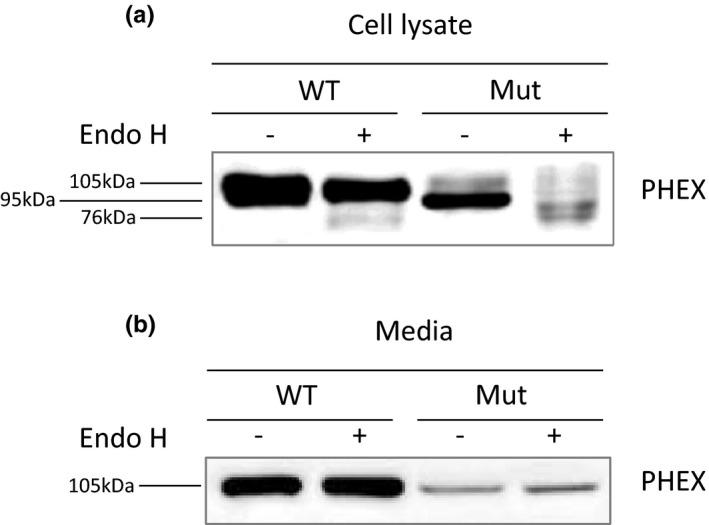
Sensibility of PHEX protein to the digestion of Endo H. (a and b) Whole‐cell lysates (a) and concentrated media (b) from HEK293 cells transiently transfected with WT or mutant PHEX vector were fractionated on 12% SDS–PAGE and analyzed by western blotting with anti‐flag antibodies. Samples were treated with or without Endo H before electrophoresis. The 105, 95, and 76 kDa bands represent mature, core‐glycosylated and deglycosylated forms of PHEX protein, respectively

Western blot results showed that the main band of mutant PHEX protein in total cell lysate was lower than that of wild‐type PHEX protein (Figure 3a), so we detected the glycosylation degree of wild‐type and mutant PHEX protein to identify the cause of this difference. Compared with untreated WT PHEX protein, the main band of WT PHEX protein treated with Endo H did not vary significantly, but a weak 76 kDa band appeared. Compared with the untreated mutant PHEX protein, the main band of the mutant PHEX protein treated with Endo H changed to 76 kDa (Figure 4a). After Endo H treatment, the bands of secreted WT PHEX (WT‐secPHEX) and secreted mutant PHEX (Mut‐secPHEX) in the cell medium showed no significant changes; that is, digested Mut‐secPHEX protein moved equally to the other three samples, so the molecular weights of all four secPHEX protein samples were 105 kDa (Figure 4b). These data indicated that the secreted WT and mutant PHEX proteins are both resistant to the digestion of mutant PHEX protein. Because Endo H can only digest core‐glycosylated proteins, these results suggested that the 76 kDa species of cellular mutant PHEX protein represent the deglycosylated core‐glycosylated forms within the ERs, the 95 kDa species of cellular PHEX protein represent the core‐glycosylated and unmatured form within the ERs, while the 105 kDa species of PHEX protein represent the fully processed, matured form (Figure 4a,b).

### 3.7 The level of intact FGF23 was elevated in the mutant PHEX transfected group

**Figure 5:**
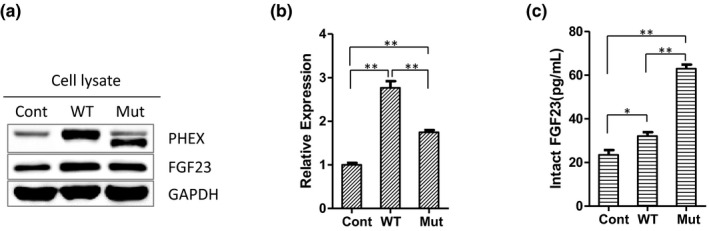
The mutant PHEX altered the expression of FGF23. (a) WT and mutant PHEX protein were both overexpressed in hFOB1.19 cells transfected with vectors coding WT PHEX (WT group) and mutant PHEX (Mut group), respectively. There is also a minor naturally expressed WT PHEX band in the blank vector‐transfected hFOB1.19 cells (Cont group). (b) RT‐qPCR analysis showed that the mRNA levels of FGF23 were both elevated in the WT and Mut groups compared to the control group. (c) ELISA analysis showed that the intact FGF23 concentration in cell culture medium was both elevated in the Mut and WT groups, especially in the Mut group. *p < .05; **p < .01. Bars show SD (*n* = 4)

Western blot analysis showed that the expression of PHEX protein and total FGF23 in the WT PHEX group and mutant PHEX group were increased significantly compared to the control group, and the expression of total FGF23 in the WT PHEX group was higher than that in the mutant PHEX group (Figure 5a). The expression levels of FGF23 mRNA in hFOB1.19 cells were measured by RT‐qPCR 24 h after transfection. The results showed that the expression levels of FGF23 mRNA in the WT and mutant PHEX groups were 2.8 times and 1.7 times higher than that in the blank control group, respectively (Figure 5b). The concentration of intact FGF23 in hFOB1.19 cell culture medium was detected by ELISA. The concentration of intact FGF23 in the supernatant of culture medium collected from the mutant PHEX group was the highest, with values of 62.9 pg/ml, while the values in the WT group and control group were much lower, which were 32.1 and 23.5 pg/ml, respectively (Figure 5c). These data indicate that the mutant PHEX protein cannot fully exert the endopeptidase activity to cleave FGF23 into an inactive form, leading to the higher level of intact FGF23 in the media supernatants.

## 4 DISCUSSION

In the present study, we identified a novel missense mutation c.2179T>C in the *PHEX* from an XLH family with two affected individuals. This mutation is highly conserved through the alignment of the PHEX protein sequences from 10 different species and is inferred to be pathogenic by all four bioinformatics tools (Adzhubei et al., 2010; Feng, 2017). The clinical phenotypes of the patients, such as high levels of intact circulating FGF23, hypophosphatemia, and severe genu varum, are consistent with the loss‐of‐function genotype of PHEX. Through immunofluorescence staining, we demonstrated that the c.2179T>C (p.F727L) mutation in the *PHEX* obstructed cytoplasmic membrane localization of the exogenous expressed mutant protein in HEK293 cells. These data are similar to previous studies of c.2158G>T (p.A720S) mutation in *PHEX* identified in a sporadic case of adult‐onset female XLH (Goljanek‐Whysall et al., 2018). Although both of them are heterozygous, the clinical features of the female XLH patient in Katarzyna's study were different from those of the proband's mother in our study. The latter is basically normal and has no pain and stiffness in the lumbar back, hips, and feet. This phenomenon indicates that the degree of function loss of the PHEX protein caused by the c.2179T>C mutation is smaller than that of the c.2158G>T mutation.

We also provide evidence that the mutant F727L PHEX protein is more sensitive to Endo H digestion compared with the WT PHEX protein, indicating that the mutant protein is not fully glycosylated and probably restricted in the ER, which may explain why only ~12% of the mutant is localized at the membrane. In addition, the mutant F727L secPHEX protein is also detected in the cell culture medium secreted by HEK293 incubated in normal conditions (37℃, 5% CO_2_), which indicated that a small part of the mutant PHEX protein may perform endopeptidase activity out of cells. A previous study showed that the mutant F731Y PHEX was normally secreted at 37℃, and the A720T mutant could only be secreted at a lower temperature 26℃ but at 37℃, both of which exhibited ~90% wild‐type endopeptidase activity (Sabbagh et al., 2003). Therefore, we inferred that the F727L mutant, which resides in the same exon (exon 22) as F731Y and A720T, may also have a similar activity level as F731Y and A720T mutants. These data may further explain why the proband's BMD is normal in adulthood. However, the real activity of the F727L PHEX protein needs further study. Because only a few mutant F727L PHEX proteins were localized on the membrane or secreted out of cells, the level of intact FGF23 in the medium supernatants of hFOB1.19 cells transfected with the mutant PHEX vector was much higher than that in the WT group, which is coordinated to the high level of intact circulating FGF23 in XLH patients.

In summary, our study demonstrated that the F727L mutation decreased the glycosylation level of PHEX protein, blocked PHEX protein transport from the ER to the plasma membrane, and reduced its activity to cleave FGF23 into inactive segments, which may explain why the single base substitution c.2179T>C led to the hypophosphatemic rickets in this Chinese family. Our findings expanded the mutation spectrum of the *PHEX* and provided a genetic basis for the prenatal or postpartum diagnosis of XLH in the future.

## CONFLICT OF INTEREST

The authors declare that they have no conflicts of interest.

## AUTHOR CONTRIBUTIONS

Baowei Li designed and completed most of the molecular cytobiological experiments. Xiong Wang provided clinical samples and family members' clinical data. Baowei Li and Xiong Wang completed the manuscript together and contributed equally to this work. Xiaodan Hao completed the Sanger sequencing experiments. Yanran Liu completed the qPCR experiments. Chan Shan and Xiang Ao helped complete plasmids construction. Yin Wang, Ying Liu, Hong Chu Bao, and Peifeng Li revised the manuscript and provided financial support.

## Supporting information

Supplementary Material

Fig S1

## Data Availability

All data included in this study are available upon request by contact with corresponding authors.

## References

[mgg31262-bib-0001] Adzhubei, I. A. , Schmidt, S. , Peshkin, L. , Ramensky, V. E. , Gerasimova, A. , Bork, P. , … Sunyaev, S. R. (2010). A method and server for predicting damaging missense mutations. Nature Methods, 7(4), 248–249. 10.1038/nmeth0410-248 20354512 PMC2855889

[mgg31262-bib-0002] Bowe, A. E. , Finnegan, R. , Jan de Beur, S. M. , Cho, J. , Levine, M. A. , Kumar, R. , & Schiavi, S. C. (2001). FGF‐23 inhibits renal tubular phosphate transport and is a PHEX substrate. Biochemical and Biophysical Research Communications, 284(4), 977–981. 10.1006/bbrc.2001.5084 11409890

[mgg31262-bib-0003] Chandran, M. , Chng, C. L. , Zhao, Y. , Bee, Y. M. , Phua, L. Y. , & Clarke, B. L. (2010). Novel *PHEX* gene mutation associated with X linked hypophosphatemic rickets. Nephron Physiology, 116(3), 17–21. 10.1159/000319318 20664300

[mgg31262-bib-0004] Clausmeyer, S. , Hesse, V. , Clemens, P. C. , Engelbach, M. , Kreuzer, M. , Becker‐Rose, P. , … Raue, F. (2009). Mutational analysis of the *PHEX* gene: Novel point mutations and detection of large deletions by MLPA in patients with X‐linked hypophosphatemic rickets. Calcified Tissue International, 85(3), 211–220. 10.1007/s00223-009-9260-8 19513579

[mgg31262-bib-0005] den Dunnen, J. T. , Dalgleish, R. , Maglott, D. R. , Hart, R. K. , Greenblatt, M. S. , McGowan‐Jordan, J. , … Taschner, P. E. M. (2016). HGVS Recommendations for the Description of Sequence Variants: 2016 Update. Human Mutation, 37(6), 564–569. 10.1002/humu.22981 26931183

[mgg31262-bib-0006] Dhir, G. , Li, D. , Hakonarson, H. , & Levine, M. A. (2017). Late‐onset hereditary hypophosphatemic rickets with hypercalciuria (HHRH) due to mutation of SLC34A3/NPT2c. Bone, 97, 15–19. 10.1016/j.bone.2016.12.001 27939817 PMC5367968

[mgg31262-bib-0007] Drezner, M. K. (2000). *PHEX* gene and hypophosphatemia. Kidney International, 57(1), 9–18. 10.1046/j.1523-1755.2000.00807.x 10620182

[mgg31262-bib-0008] Durmaz, E. , Zou, M. , Al‐Rijjal, R. A. , Baitei, E. Y. , Hammami, S. , Bircan, İ. , … Shi, Y. (2013). Novel and de novo *PHEX* mutations in patients with hypophosphatemic rickets. Bone, 52(1), 286–291. 10.1016/j.bone.2012.10.012 23079138

[mgg31262-bib-0009] Feng, B. J. (2017). PERCH: A unified framework for disease gene prioritization. Human Mutation, 38(3), 243–251. 10.1002/humu.23158 27995669 PMC5299048

[mgg31262-bib-0010] Fuente, R. , Gil‐Peña, H. , Claramunt‐Taberner, D. , Hernández, O. , Fernández‐Iglesias, A. , Alonso‐Durán, L. , … Santos, F. (2017). X‐linked hypophosphatemia and growth. Reviews in Endocrine & Metabolic Disorders, 18(1), 107–115. 10.1007/s11154-017-9408-1 28130634

[mgg31262-bib-0011] Goljanek‐Whysall, K. , Tridimas, A. , McCormick, R. , Russell, N.‐J. , Sloman, M. , Sorani, A. , … Hannan, F. M. (2018). Identification of a novel loss‐of‐function PHEX mutation, Ala720Ser, in a sporadic case of adult‐onset hypophosphatemic osteomalacia. Bone, 106, 30–34. 10.1016/j.bone.2017.10.002 28982589

[mgg31262-bib-0012] Guo, R. , & Quarles, L. D. (1997). Cloning and sequencing of human PEX from a bone cDNA library: Evidence for its developmental stage‐specific regulation in osteoblasts. Journal of Bone and Mineral Research, 12(7), 1009–1017. 10.1359/jbmr.1997.12.7.1009 9199999

[mgg31262-bib-0013] Hu, J. L. , Hua, Y. J. , Chen, Y. , Yu, B. , & Gao, S. (2015). Structural analysis of tumor‐related single amino acid mutations in human MxA protein. Chinese Journal of Cancer, 34(12), 583–593. 10.1186/s40880-015-0055-1 26411585 PMC4593380

[mgg31262-bib-0014] Kapelari, K. , Kohle, J. , Kotzot, D. , & Hogler, W. (2015). Iron supplementation associated with loss of phenotype in autosomal dominant hypophosphatemic rickets. Journal of Clinical Endocrinology and Metabolism, 100(9), 3388–3392. 10.1210/jc.2015-2391 26186302

[mgg31262-bib-0015] Li, B. , Wang, S. , Liu, H. , Liu, D. , Zhang, J. , Zhang, B. , … Pei, X. (2011). Neuronal restrictive silencing factor silencing induces human amniotic fluid‐derived stem cells differentiation into insulin‐producing cells. Stem Cells and Development, 20(7), 1223–1231. 10.1089/scd.2010.0195 20942606

[mgg31262-bib-0016] Lipman, M. L. , Panda, D. , Bennett, H. P. , Henderson, J. E. , Shane, E. , Shen, Y. , … Karaplis, A. C. (1998). Cloning of human PEX cDNA. Expression, subcellular localization, and endopeptidase activity. Journal of Biological Chemistry, 273(22), 13729–13737. 10.1074/jbc.273.22.13729 9593714

[mgg31262-bib-0017] Pal, R. , Bhadada, S. K. , Shingare, A. , Bhansali, A. , Kamalanathan, S. , Chadha, M. , … Agashe, V. (2019). Tumor‐induced osteomalacia: Experience from three tertiary care centres In India. Endocrine Connections, 8(3), 266–276. 10.1530/ec-18-0552 30726771 PMC6410764

[mgg31262-bib-0018] Quarles, L. D. (2012). Skeletal secretion of FGF‐23 regulates phosphate and vitamin D metabolism. Nature Reviews Endocrinology, 8(5), 276–286. 10.1038/nrendo.2011.218 PMC4489419 22249518

[mgg31262-bib-0019] Quarles, L. D. , & Drezner, M. K. (2001). Pathophysiology of X‐linked hypophosphatemia, tumor‐induced osteomalacia, and autosomal dominant hypophosphatemia: A perPHEXing problem. Journal of Clinical Endocrinology and Metabolism, 86(2), 494–496. 10.1210/jcem.86.2.7302 11157997

[mgg31262-bib-0020] Quinlan, C. , Guegan, K. , Offiah, A. , Neill, R. O’. , Hiorns, M. P. , Ellard, S. , … Waters, A. M. (2012). Growth in PHEX‐associated X‐linked hypophosphatemic rickets: The importance of early treatment. Pediatric Nephrology, 27(4), 581–588. 10.1007/s00467-011-2046-z 22101457

[mgg31262-bib-0021] Rowe, P. S. (1994). Molecular biology of hypophosphataemic rickets and oncogenic osteomalacia. Human Genetics, 94(5), 457–467. 10.1007/bf00211008 7959677

[mgg31262-bib-0022] Rowe, P. S. (1998). The role of the *PHEX* gene (PEX) in families with X‐linked hypophosphataemic rickets. Current Opinion in Nephrology and Hypertension, 7(4), 367–376. 10.1097/00041552-199807000-00004 9690034

[mgg31262-bib-0023] Ruppe, M. D. , Brosnan, P. G. , Au, K. S. , Tran, P. X. , Dominguez, B. W. , & Northrup, H. (2011). Mutational analysis of *PHEX*, *FGF23* and *DMP1* in a cohort of patients with hypophosphatemic rickets. Clinical Endocrinology ‐ Oxford, 74(3), 312–318. 10.1111/j.1365-2265.2010.03919.x 21050253 PMC3035757

[mgg31262-bib-0024] Sabbagh, Y. , Boileau, G. , Campos, M. , Carmona, A. K. , & Tenenhouse, H. S. (2003). Structure and function of disease‐causing missense mutations in the PHEX gene. Journal of Clinical Endocrinology and Metabolism, 88(5), 2213–2222. 10.1210/jc.2002-021809 12727977

[mgg31262-bib-0025] Sabbagh, Y. , Boileau, G. , DesGroseillers, L. , & Tenenhouse, H. S. (2001). Disease‐causing missense mutations in the *PHEX* gene interfere with membrane targeting of the recombinant protein. Human Molecular Genetics, 10(15), 1539–1546. 10.1093/hmg/10.15.1539 11468271

[mgg31262-bib-0026] Yamazaki, Y. , Okazaki, R. , Shibata, M. , Hasegawa, Y. , Satoh, K. , Tajima, T. , … Fukumoto, S. (2002). Increased circulatory level of biologically active full‐length FGF‐23 in patients with hypophosphatemic rickets/osteomalacia. Journal of Clinical Endocrinology and Metabolism, 87(11), 4957–4960. 10.1210/jc.2002-021105 12414858

[mgg31262-bib-0027] Yue, H. , Yu, J.‐B. , He, J.‐W. , Zhang, Z. , Fu, W.‐Z. , Zhang, H. , … Zhang, Z.‐L. (2014). Identification of two novel mutations in the *PHEX* gene in Chinese patients with hypophosphatemic rickets/osteomalacia. PLoS ONE, 9(5), e97830 10.1371/journal.pone.0097830 24836714 PMC4024000

