# Supplementary material for: A novel c.2179T>C mutation blocked the intracellular transport of PHEX protein and caused X‐linked hypophosphatemic rickets in a Chinese family
Source: Mol Genet Genomic Med. 2020 Jun 8;8(8):e1262. doi: 10.1002/mgg3.1262 (PMC7434742; doi:10.1002/mgg3.1262)

| Table S1. BMD of the proband | | | | |
| --- | --- | --- | --- | --- |
| Region | BMD (g/cm2) | Compared with healthy young adults (T value) | Compared with normal contemporaries (Z value) | ISCD diagnostic criteria |
| L1-L3 | 1.576 | 4.2 | 4.1 | BMD is in the range of normal contemporaries (Z > -2);  BMD is lower than normal contemporaries (Z ≤ -2) |
| Left femoral neck | 1.252 | 2.1 | 2.2 |  |
| Right femoral neck | 1.112 | 1 | 1.2 |  |
| All left hip joint | 1.32 | 2.5 | 2.5 |  |
| All right hip joint | 1.254 | 2 | 2 |  |

| Table S2. Serum intact human FGF23 levels in healthy controls | | | | | | | | | |
| --- | --- | --- | --- | --- | --- | --- | --- | --- | --- |
| Subjects | FGF23 (pg/ml) | Subjects | FGF23 (pg/ml) | Subjects | FGF23 (pg/ml) | Subjects | FGF23 (pg/ml) | Subjects | FGF23 (pg/ml) |
| 1 | 32.5 | 21 | 51.2 | 41 | 42.7 | 61 | 54.2 | 81 | 35.9 |
| 2 | 46.1 | 22 | 37.2 | 42 | 34.1 | 62 | 35.1 | 82 | 53.2 |
| 3 | 35.7 | 23 | 32.6 | 43 | 35.2 | 63 | 45.2 | 83 | 34.2 |
| 4 | 53.8 | 24 | 56.3 | 44 | 55.9 | 64 | 36.7 | 84 | 26.2 |
| 5 | 35.1 | 25 | 45.8 | 45 | 60.2 | 65 | 53.7 | 85 | 44.6 |
| 6 | 28.6 | 26 | 46.2 | 46 | 47.1 | 66 | 35.2 | 86 | 36.2 |
| 7 | 44.3 | 27 | 33.8 | 47 | 36.4 | 67 | 61.4 | 87 | 38.9 |
| 8 | 37.5 | 28 | 51.3 | 48 | 38.1 | 68 | 46.7 | 88 | 35.1 |
| 9 | 45.2 | 29 | 45.9 | 49 | 42.1 | 69 | 52.4 | 89 | 44.2 |
| 10 | 43.6 | 30 | 52.4 | 50 | 44.7 | 70 | 51.7 | 90 | 46.3 |
| 11 | 66.4 | 31 | 42.6 | 51 | 52.1 | 71 | 35.2 | 91 | 37.5 |
| 12 | 43.6 | 32 | 33.7 | 52 | 23.7 | 72 | 28.4 | 92 | 51.6 |
| 13 | 36.5 | 33 | 46.3 | 53 | 56.3 | 73 | 33.6 | 93 | 49.3 |
| 14 | 35.8 | 34 | 41.3 | 54 | 36.5 | 74 | 42.6 | 94 | 46.5 |
| 15 | 29.4 | 35 | 53.1 | 55 | 46.1 | 75 | 53.1 | 95 | 37.4 |
| 16 | 39.2 | 36 | 46.8 | 56 | 26.5 | 76 | 34.7 | 96 | 34.1 |
| 17 | 52.9 | 37 | 49.2 | 57 | 36.2 | 77 | 52.4 | 97 | 26.4 |
| 18 | 38.8 | 38 | 31.6 | 58 | 55.2 | 78 | 39.5 | 98 | 36.8 |
| 19 | 46.8 | 39 | 36.5 | 59 | 48.3 | 79 | 42.4 | 99 | 28.5 |
| 20 | 56.2 | 40 | 57.2 | 60 | 56.2 | 80 | 36.5 | 100 | 35.8 |

**Fig. S1. BMD analysis of the proband by DEXA.** (A) The X-ray image of the proband's vertebrae showing the first to the fourth lumbar vertebrae (L1-L4). (B) The BMD of L1-L3 is 1.576 g/cm2, and the T value is 4.2, which indicates that the BMD of the proband's lumbar spine is in the normal range. (C) The double femoral X-ray image of the proband showed the checked location of BMD in the lower limbs, the femoral neck. (D) The BMD of the left femoral neck was 1.252 g/cm2 and that of the right femoral neck was 1.112 g/cm2. The T values were 2.1 and 1.0, respectively. These data indicated that the BMD of the proband's femoral neck was in the normal range.


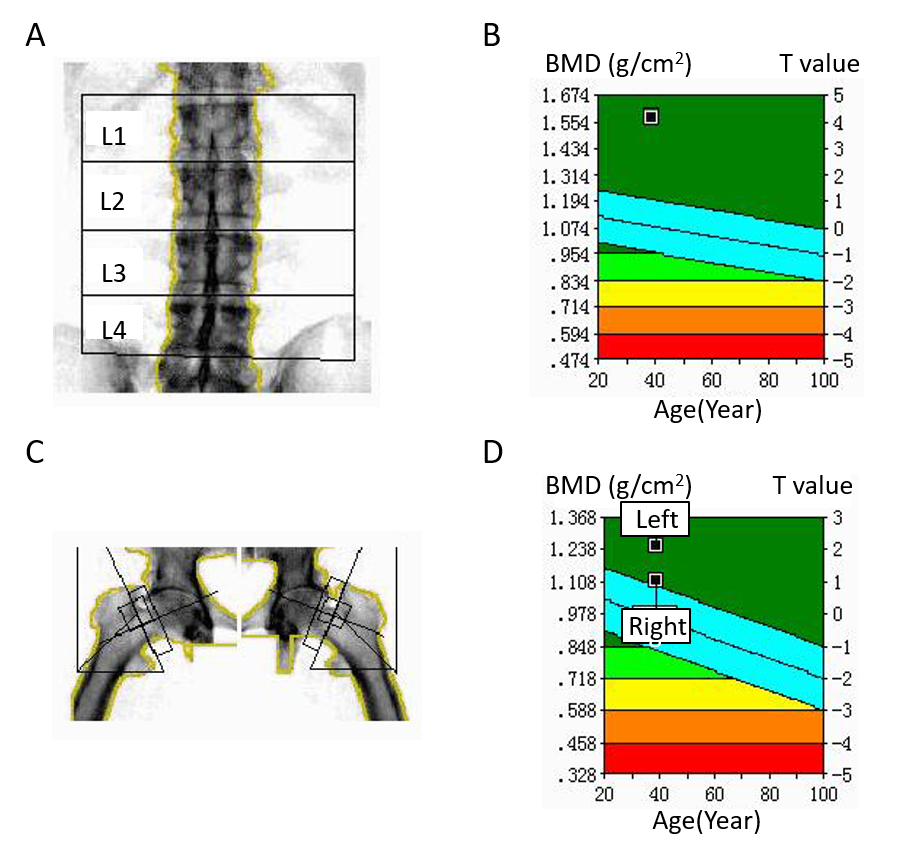

Supplement: Supplementary file 1 — Supplementary Material [file MGG3-8-e1262-s001.docx]
