# Supplementary figures and images for: A novel c.2179T>C mutation blocked the intracellular transport of PHEX protein and caused X‐linked hypophosphatemic rickets in a Chinese family
Source: Mol Genet Genomic Med. 2020 Jun 8;8(8):e1262. doi: 10.1002/mgg3.1262 (PMC7434742; doi:10.1002/mgg3.1262)

A

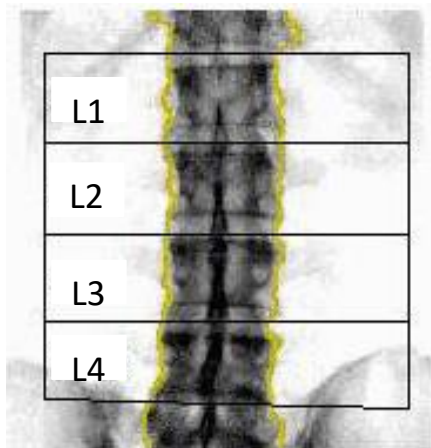

B

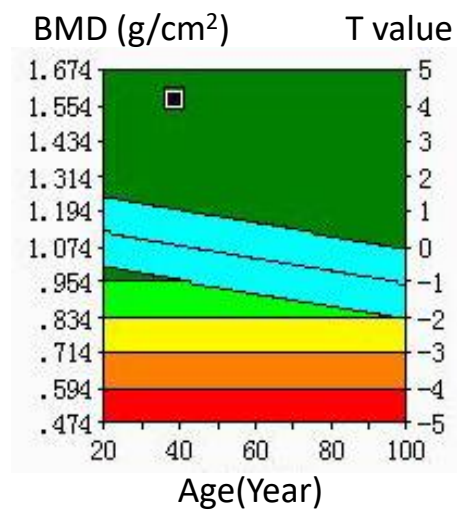

C

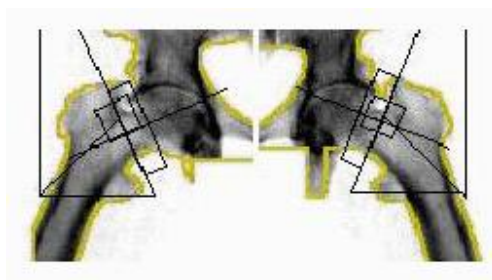

D

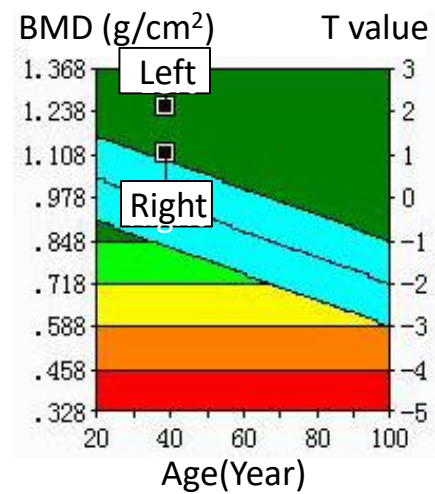

Supplement: Supplementary file 2 — Fig S1 [file MGG3-8-e1262-s002.pdf]
